# Naturally occurring mitochondrial-derived peptides are age-dependent regulators of apoptosis, insulin sensitivity, and inflammatory markers

**DOI:** 10.18632/aging.100943

**Published:** 2016-04-10

**Authors:** Laura J. Cobb, Changhan Lee, Jialin Xiao, Kelvin Yen, Richard G. Wong, Hiromi K. Nakamura, Hemal H. Mehta, Qinglei Gao, Carmel Ashur, Derek M. Huffman, Junxiang Wan, Radhika Muzumdar, Nir Barzilai, Pinchas Cohen

**Affiliations:** ^1^ Department of Pediatrics, Mattel Children's Hospital, and Division of Endocrinology, David Geffen School of Medicine, University of California, Los Angeles, CA 90095, USA; ^2^ Leonard Davis School of Gerontology, University of Southern California, Los Angeles, CA 90089, USA; ^3^ Department of Pediatrics, Children's Hospital of Pittsburgh, Pittsburgh, PA 15224, USA; ^4^ Department of Medicine, Albert Einstein College of Medicine, Bronx, NY 10461, USA; ^5^ Current address: LCS Executive Consulting, North Hollywood, CA 91607, USA

**Keywords:** humanin, SHLP, aging, mitochondria, small ORFs

## Abstract

Mitochondria are key players in aging and in the pathogenesis of age-related diseases. Recent mitochondrial transcriptome analyses revealed the existence of multiple small mRNAs transcribed from mitochondrial DNA (mtDNA). Humanin (HN), a peptide encoded in the mtDNA 16S ribosomal RNA region, is a neuroprotective factor. An *in silico* search revealed six additional peptides in the same region of mtDNA as humanin; we named these peptides small humanin-like peptides (SHLPs). We identified the functional roles for these peptides and the potential mechanisms of action. The SHLPs differed in their ability to regulate cell viability *in vitro*. We focused on SHLP2 and SHLP3 because they shared similar protective effects with HN. Specifically, they significantly reduced apoptosis and the generation of reactive oxygen species, and improved mitochondrial metabolism *in vitro*. SHLP2 and SHLP3 also enhanced 3T3-L1 pre-adipocyte differentiation. Systemic hyperinsulinemic-euglycemic clamp studies showed that intracerebrally infused SHLP2 increased glucose uptake and suppressed hepatic glucose production, suggesting that it functions as an insulin sensitizer both peripherally and centrally. Similar to HN, the levels of circulating SHLP2 were found to decrease with age. These results suggest that mitochondria play critical roles in metabolism and survival through the synthesis of mitochondrial peptides, and provide new insights into mitochondrial biology with relevance to aging and human biology.

## INTRODUCTION

Human mitochondrial DNA (mtDNA) is a double-stranded, circular molecule of 16,569 bp and contains 37 genes encoding 13 proteins, 22 tRNAs, and 2 rRNAs. Recent mitochondrial transcriptome analyses revealed the existence of small RNAs derived from mtDNA [1]. In 2001, Nishimoto and colleagues identified humanin (HN), a 24-amino-acid peptide encoded from the 16S ribosomal RNA (rRNA) region of mtDNA. HN is a potent neuroprotective factor capable of antagonizing Alzheimer's disease (AD)-related cellular insults [2]. HN is a component of a novel retrograde signaling pathway from the mitochondria to the nucleus, which is distinct from mitochondrial signaling pathways, such as the SIRT4-AMPK pathway [3]. HN-dependent cellular protection is mediated in part by interacting with and antagonizing pro-apoptotic Bax-related peptides [4] and IGFBP-3 (IGF binding protein 3) [5].

Because of their involvement in energy production and free radical generation, mitochondria likely play a major role in aging and age-related diseases [6–8]. In fact, improvement of mitochondrial function has been shown to ameliorate age-related memory loss in aged mice [9]. Recent studies have shown that HN levels decrease with age, suggesting that HN could play a role in aging and age-related diseases, such as Alzheimer's disease (AD), atherosclerosis, and diabetes. Along with lower HN levels in the hypothalamus, skeletal muscle, and cortex of older rodents, the circulating levels of HN were found to decline with age in both humans and mice [10]. Notably, circulating HN levels were found to be (i) significantly higher in long-lived Ames dwarf mice but lower in short-lived growth hormone (GH) transgenic mice, (ii) significantly higher in a GH-deficient cohort of patients with Laron syndrome, and (iii) reduced in mice and humans treated with GH or IGF-1 (insulin-like growth factor 1) [11]. Age-dependent declines in the circulating HN levels may be due to higher levels of reactive oxygen species (ROS) that contribute to atherosclerosis development. Using mouse models of atherosclerosis, it was found that HN-treated mice had a reduced disease burden and significant health improvements [12, 13]. In addition, HN improved insulin sensitivity, suggesting clinical potential for mitochondrial peptides in diseases of aging [10]. The discovery of HN represents a unique addition to the spectrum of roles that mitochondria play in the cell [14, 15]. A second mitochondrial-derived peptide (MDP), MOTS-c (mitochondrial open reading frame of the 12S rRNA-c), has also been shown to have metabolic effects on muscle and may also play a role in aging [16].

We further investigated mtDNA for the presence of other MDPs. Recent technological advances have led to the identification of small open reading frames (sORFs) in the nuclear genomes of *Drosophila* [17, 18] and mammals [19, 20]. Therefore, we attempted to identify novel sORFs using the following approaches: 1) *in silico* identification of potential sORFs; 2) determination of mRNA expression levels; 3) development of specific antibodies against these novel peptides to allow for peptide detection in cells, organs, and plasma; 4) elucidating the actions of these peptides by performing cell-based assays for mitochondrial function, signaling, viability, and differentiation; and 5) delivering these peptides *in vivo* to determine their systemic metabolic effects. Focusing on the 16S rRNA region of the mtDNA where the *humanin* gene is located, we identified six sORFs and named them small humanin-like peptides (SHLPs) 1–6. While surveying the biological effects of SHLPs, we found that SHLP2 and SHLP3 were cytoprotective; therefore, we investigated their effects on apoptosis and metabolism in greater detail. Further, we showed that circulating SHLP2 levels declined with age, similar to HN, suggesting that SHLP2 is involved in aging and age-related disease progression.

## RESULTS

### Identification and validation of sORFs within mitochondrial 16S rRNA

**Figure 1:**
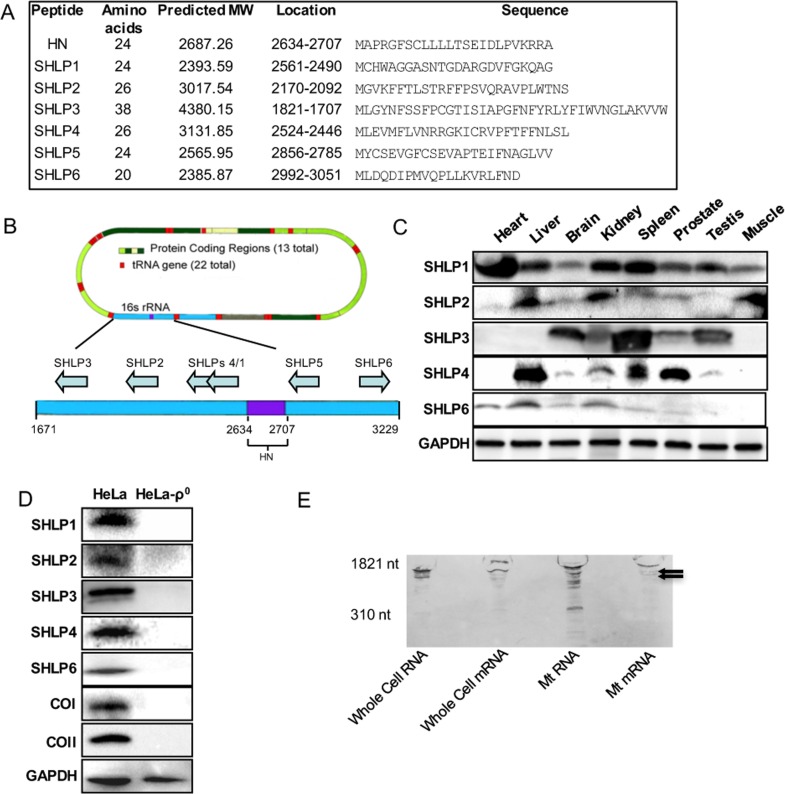
Identification and validation of small open reading frames (sORFs) within the mitochondrial 16S ribosomal RNA (rRNA) gene (**A**) Assigned names, size, location, and predicted sequences of SHLPs. (**B**) Location of SHLP ORFs within 16S rRNA. (**C**) Relative expression of SHLPs (1–4 and 6) in C57BL/6 mouse tissues relative to GAPDH (used as a loading control). (**D**) Expression of SHLPs (1–4 and 6), mitochondrial complexes (COI and COII) and nuclear GAPDH proteins in HeLa parental and HeLa-ρ0 cells. (**E**) Northern blot of whole cell RNA, whole cell mRNA, mitochondrial RNA, and mitochondrial mRNA using a SHLP6 probe identifies several bands smaller than the 16S rRNA indicated by the arrows.

An *in silico* search for potential sORFs within 16S-rRNA-encoding short peptides (20–40 amino acids) returned six sequences encoding 20–38 amino-acid-long peptides (Fig. 1A, B), which we named SHLP 1–6. To assess potential endogenous expression, antibodies against SHLPs 1, 2, 3, 4, and 6 were generated; immunoblotting was used to assess SHLP expression in mouse tissues including the heart, liver, brain, kidney, spleen, prostate, testis, and skeletal muscle. Despite multiple attempts, we were unable to obtain a specific antibody against SHLP5. Multiple mouse tissues expressed SHLPs 1–4 and 6 at varying levels (Fig. 1C). We observed organ-specific expression of individual SHLPs. Specifically, SHLP1 was detected in the heart, kidney, and spleen; SHLP2 was detected in the liver, kidney, and muscle; SHLP3 was detected in the brain and spleen; SHLP4 was detected in the liver and prostate; and SHLP6 was detected in the liver and kidney.

The *humanin* gene is fully homologous to a region of the mitochondrial 16S rRNA [2, 4, 5], and shares 92–95% homology with several regions in the nuclear genome, reflecting a phenomena known as nuclear mitochondrial DNA transfer (NUMT) [21]. Therefore, we used the Basic Local Alignment Search Tool (BLAST; http://blast.ncbi.nlm.nih.gov/Blast.cgi) to search for nuclear homologues of SHLPs and identified several potential nuclear isoforms. To identify sites of SHLP expression, we isolated highly purified mRNA from human prostate cancer cells (22Rv1) and amplified and sequenced either mitochondrial or nuclear isoforms of SHLPs 1–6 using specifically designed primers (Table S1). Both the reverse transcription polymerase chain reaction (RT-PCR) and sequencing data showed that the sORFs for SHLPs 1, 4, 5, and 6 were mitochondrial, not nuclear, in origin (Fig. S1). SHLP2 and SHLP3 were amplified from both mitochondrial and nuclear cDNA; therefore, RT-PCR by itself was inconclusive for determining their origins (Fig. S1).

To further confirm the mitochondrial origin of SHLPs, we compared the expression levels of SHLPs 1–4 and 6 in parental HeLa cells and HeLa cells selectively devoid of mitochondrial DNA (HeLa-ρ0) [42]. SHLPs 1–4 and 6 and the mitochondrial-encoded proteins cytochrome oxidase I and II (COI and COII) were detected in parental HeLa cells, but not in HeLa-ρ0 cells (Fig. 1D). This suggests a mitochondrial origin of these peptides; however, we could not completely rule out the possibility that these effects were secondary to the loss of mitochondria. To provide further support for the mitochondrial origin of the SHLP peptides, we used Northern blotting to probe for the presence of total and polyadenylated mRNA transcripts from the 16S rRNA region of mtDNA from (a) whole cell total RNA, (b) whole cell mRNA, (c) mitochondrial total RNA, and (d) mitochondrial mRNA (Fig. 1E). We detected many mitochondrial mRNA transcripts of various sizes that were smaller than the full-length rRNA transcript, suggesting the presence of mRNA species corresponding to specific regions of the 16S region (Fig. 1E) that encode SHLPs. Together, these data support that SHLP sORFs are mitochondrial in origin.

### SHLPs are bioactive peptides that modulate cell function

**Figure 2:**
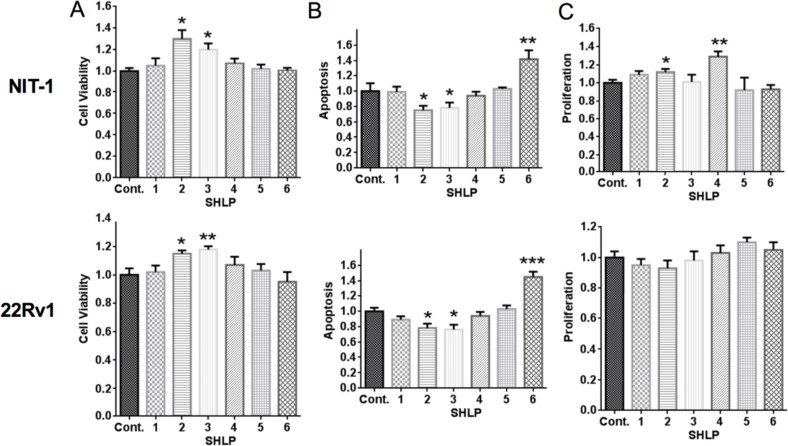
Effect of SHLPs on cell growth and death NIT-1 β and 22Rv1 cells were cultured in serum-free (SF) media with 100 nM SHLP or control peptides and assessed for (**A**) cell viability (using the MTS assay) after 72 h; (**B**) apoptosis after 24 h; and (**C**) cell proliferation by BrdU incorporation after 24 h. All data are presented as means ± SEM, and significance was determined by Student's *t*-tests. *P < 0.05; **P < 0.01; ***P < 0.001.

After assessing the expression of endogenous SHLPs, we next determined their physiological roles. Murine β-cells (NIT-1) and human prostate cancer cells (22Rv1) were incubated with control peptide or SHLPs 1–6 under serum-starved conditions. Cell viability was assessed by an MTS ([3-(4,5-dimethylthiazol-2-yl)-5-(3-carboxymethoxyphenyl)-2-(4-sulfophenyl)-2H-tetra-zolium) assay, apoptosis was assessed by DNA fragmentation, and cellular proliferation was assessed by BrdU (bromodeoxyuridine) incorporation. SHLP2 and SHLP3 enhanced cell viability (Fig. 2A) and decreased apoptosis in both NIT-1 and 22Rv1 cells (Fig. 2B). SHLP2 and SHLP4 promoted cell proliferation in NIT-1 β-cells (Fig. 2C). SHLP6 significantly increased apoptosis in both NIT-1 and 22Rv1 cells (Fig. 2B), having an effect opposite of SHLP2 and SHLP3.

### SHLP2 and SHLP3 modulate mitochondrial function

**Figure 3:**
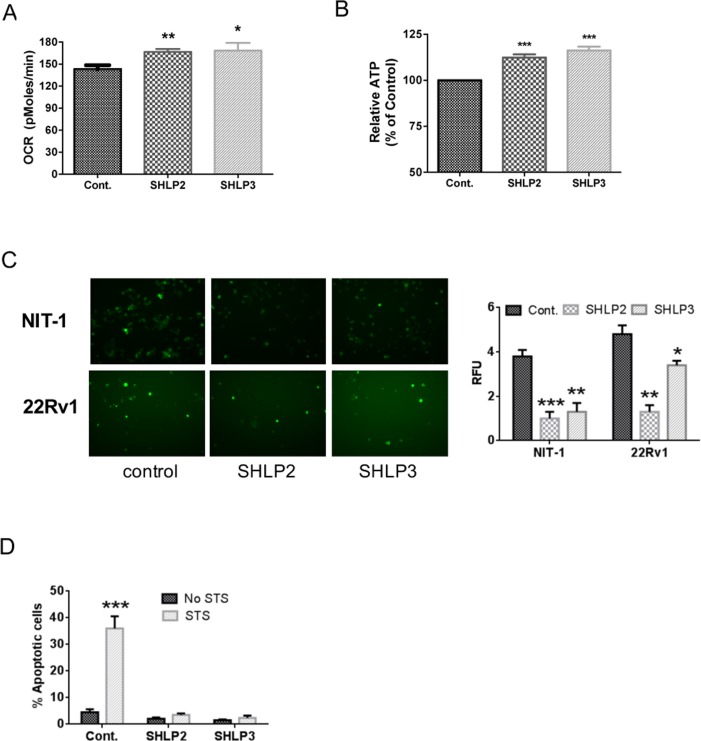
SHLP2 and SHLP3 modulate mitochondrial function The effects of exogenous SHLP2 and SHLP3 on mitochondria were assessed in 22Rv1 cells incubated with 100 nM control peptide, SHLP2, or SHLP3 for 24 h by measuring (**A**) oxygen consumption rate (OCR) performed on a Seahorse XF24 Extracellular Flux Analyzer and (**B**) ATP production. (**C**) Reactive oxygen species (ROS) production as assessed by DHE (dihydroethidium) fluorescence in NIT-1 (top) and 22RV1 (bottom) cells after incubation with 100 nM control peptide, SHLP2, or SHLP3 overnight. All data are presented as means ± SEM. (**D**) NIT-1 β cells were pre-incubated with 100 nM SHLP2 or SHLP3 for 5 h, followed by incubation with 10 μM staurosporine (STS) for 24 h. Apoptosis (pre-G1 peak) was assessed by FACS (fluorescence-activated cell sorting) analysis. *P < 0.05; **P < 0.01; ***P < 0.001.

Because SHLP2 and SHLP3 were the MDPs that induced the most robust responses in our assays, we selected them for further analysis. To assess the effect of SHLP2 and SHLP3 on cellular metabolism, we measured the mitochondrial oxygen consumption rate (OCR) in 22Rv1 cells in real time using an XF24 Extracellular Flux Analyzer. Interestingly, both SHLP2 and SHLP3 significantly increased the OCR (Fig. 3A) and cellular ATP (adenosine triphosphate) levels (Fig. 3B). Taken together, these data suggest that SHLP2 and SHLP3 enhance mitochondrial metabolism.

During oxidative stress, cellular ROS accumulation is associated with mitochondrial dysfunction [22] and aging; however, an increase in ROS may be beneficial in specific circumstances [23]. Having previously shown that HN can protect against ROS formation stimulated by oxidized low-density lipoprotein (LDL) in human aortic endothelial cells [12], we hypothesized that SHLP2 and SHLP3 may have similar effects against ROS production. Pre-incubation of NIT-1 and 22Rv1 cells with SHLP2 or SHLP3 overnight significantly suppressed serum-starvation-dependent ROS production (Fig. 3C), suggesting a cytoprotective role.

To further assess the cytoprotective effects of SHLP2 and SHLP3, we used staurosporine (STS), which causes impairment of mitochondrial membrane potential, to activate caspase-3 [24]. STS-induced apoptosis was fully blocked by SHLP2 and SHLP3 in NIT-1 β cells (Fig. 3D). Together, these results show that SHLP2 and SHLP3 affect mitochondrial function.

### Similar to HN, SHLP2 and SHLP3 induce ERK (extracellular signal-regulated kinase) and STAT-3 (signal transducer and activator of transcription 3) phosphorylation

**Figure 4:**
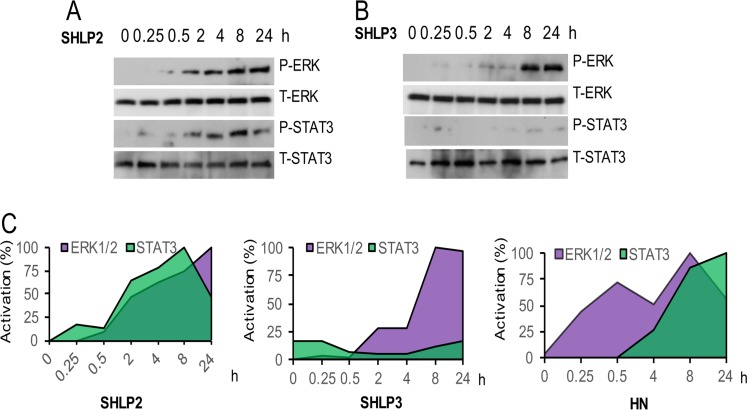
ERK and STAT-3 activation by SHLP2 and SHLP3 Levels of phospho- and total STAT3 and ERK were assessed in NIT-1 β cells treated with 100 nM (**A**) SHLP2 or (**B**) SHLP3. (**C**) Quantification of ERK and STAT3 levels after treatment with SHLP2, SHLP3, and HN. HN data were derived from Hoang et al., 2009.

Because ERK and STAT3 are phosphorylated upon HN stimulation [25], we hypothesized that the protective effect of SHLP2 and SHLP3 may also be mediated via ERK and STAT3 signaling. We observed that SHLP2 activated both ERK and STAT3 pathways in NIT-1 β cells in a time-dependent manner (Fig. 4A) but with different kinetics than those induced by HN (Fig. 4A, C). SHLP3 activated ERK at a later time point, but did not activate STAT3 phosphorylation (Fig. 4B, C), suggesting different mechanisms of protection mediated by SHLP2 and SHLP3.

### SHLP2 is an insulin sensitizer that acts both peripherally and centrally

**Figure 5:**
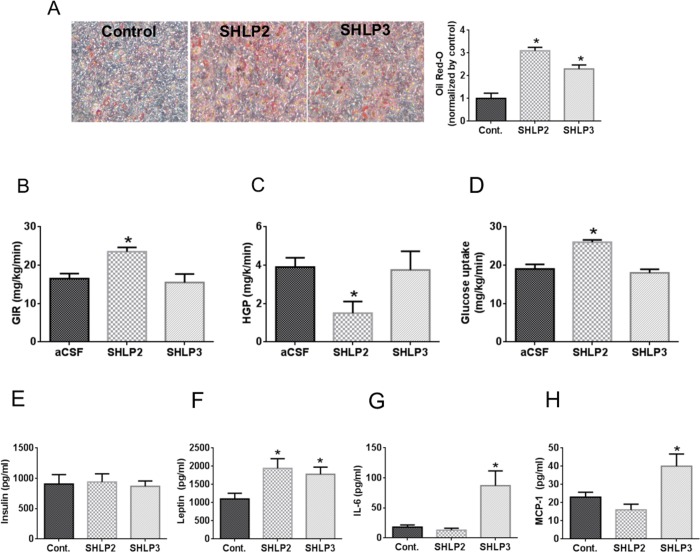
*In vitro* and *in vivo* metabolic effects of SHLP2 and SHLP3 on insulin action and pro-inflammatory biomarker expression (**A**) 3T3-L1 pre-adipocyte differentiation was assessed by Oil Red-O staining after incubation with insulin in the presence of control peptide, SHLP2, or SHLP3. (**B–D**) SHLP2 or SHLP3 were infused by ICV at a rate of 0.16 μg/kg/min into conscious SD rats (n = 6) and glucose flux was studied acutely under systemic pancreatic insulin clamp and physiologic hyperinsulinemic-euglycemic clamp. Glucose infusion rate (**B**), hepatic glucose production (**C**), and peripheral glucose uptake (**D**) were measured. (**E–F**) C57BL/6 mice (n = 5) were treated with control, SHLP2, or SHLP3 peptides (2 mg/kg/dose, BID, IP) for 5 days. Serum insulin (**E**), leptin (**F**), interleukin-6 (**G**), and monocyte chemotactic protein 1 (MCP-1) (**H**) levels were measured using LINCOplex^TM^ analysis. All data are presented as means ± SEM. *P < 0.05.

In the presence of insulin, SHLP2 and SHLP3 treatment for 7 days accelerated the differentiation of 3T3-L1 murine pre-adipocytes, as measured by Oil Red-O staining (Fig. 5A), suggesting that these peptides promote cellular differentiation and enhance insulin sensitivity in adipose tissue. Adipose tissue has been implicated in aging and age-related diseases [26]. As SHLP2 and SHLP3 increased insulin sensitivity *in vitro*, we hypothesized that they may also regulate insulin action *in vivo*. Furthermore, because of the likely connection between mitochondria, an energy generator, and the hypothalamus, an energy regulator, we assessed the metabolic consequences of centrally delivered SHLPs. Intracerebroventricular (ICV) delivery of HN regulates peripheral (hepatic) insulin action [10]; therefore, we investigated the ability of SHLP2 and SHLP3 to exert similar effects. Similar to studies done with HN, we performed systemic pancreatic insulin clamp and physiologic hyperinsulinemic-euglycemic clamp studies to quantify glucose flux [10]. SHLP2, SHLP3, and artificial cerebrospinal fluid (aCSF) were infused intracerebrally (rate, 0.16 μg/kg/min) into conscious Sprague Dawley (SD) rats to determine if SHLP2 and SHLP3 affected the hypothalamic response to peripheral insulin. SHLP2 improved insulin responsiveness as reflected by an approximately 50% increase in the exogenous glucose infusion rate (GIR) required to maintain euglycemia during insulin stimulation (Fig. 5B). The ability of insulin to suppress hepatic glucose production (HGP) (Fig. 5C) and promote glucose disposal into peripheral tissues (Fig. 5D) was enhanced by SHLP2. Unlike SHLP2, SHLP3 did not have an effect on insulin action *in vivo* (Fig. 5B–D).

Inflammation has been linked to insulin resistance [27] and increased pro-inflammatory cytokines, such as TNF-α (tumor necrosis factor α), have been shown to cause insulin resistance in experimental models [28–30]. To assess the modulating effect of SHLP2 and SHLP3 on metabolic and inflammatory profiles, we treated C57BL/6 mice with a control peptide, SHLP2, or SHLP3 for 5 days (2 mg/kg/dose, BID and examined the levels of several biomarkers by LINCOplex™. Consistent with our *in* vitro data with 3T3-L1 cells, the leptin levels increased with SHLP2 and SHLP3 treatment (Fig. 5E) with no change in body weight or food intake (data not shown). SHLP2 treatment did not alter the insulin, interleukin-6 (IL-6), or monocyte chemotactic protein-1 (MCP-1) levels (Fig. 5E–H). In contrast, SHLP3 treatment significantly increased IL-6 and MCP-1 levels, but also had no effect on insulin levels (Fig. 5E–H).

### SHLP2 plasma levels decrease with age

**Figure 6:**
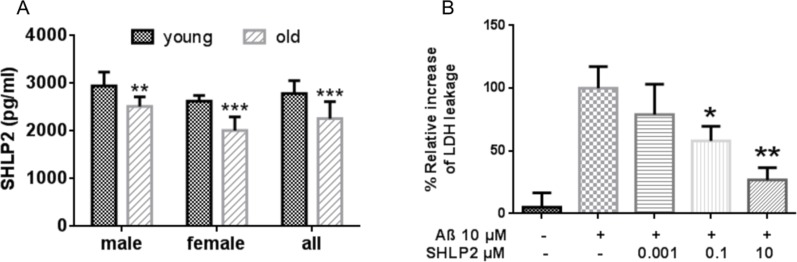
Circulating SHLP2 levels decrease with age in mice and protect against amyloid-beta (Aβ) toxicity (**A**) Plasma SHLP2 levels in young and old male and female C57BL/6 mice (n = 10 in each group) were assessed by ELISA. (**B**) Mouse primary cortical neurons were treated with vehicle control, 10 μM Aβ_1–42_ alone, or co-treated 10 μM Aβ_1–42_ with 1 nM, 100 nM, or 10 μM SHLP2. Aβ_1–42_-induced cytotoxicity was measured using a LDH (lactate dehydrogenase) cytotoxicity assay kit.

Based on the metabolic and protective roles of SHLP2 and SHLP3 *in vitro* and *in vivo*, as well as our previously published data on the age-dependency of HN and MOTS-c, we hypothesized that circulating levels of SHLP2 and SHLP3 may vary in young and old mice. We developed a novel SHLP2 immunoassay to measure the SHLP2 levels in plasma from young (3 months old) and old (18 months old) mice. We were unable to develop a suitable SHLP3 immunoassay. We found that the SHLP2 levels were significantly lower in older mice compared with younger mice (Fig. 6). The circulating SHLP2 levels significantly decreased with age in both male and female mice (Fig. 6A). Furthermore, we observed that male mice had higher SHLP2 levels than did female mice in both the young and old groups (p < 0.05).

Since SHLP2 and SHLP3 improve cell viability, have anti-apoptotic properties, and decrease (SHLP2) with age, we next sought to determine if they could help prevent neuronal death due to amyloid beta (Aβ) toxicity using an *in vitro* AD model. SHLP2, but not SHLP3, was able to prevent neuronal cell death as measured by lactate dehydrogenase (LDH) leakage when cells were exposed to Aβ (Fig. 6B). This suggests that SHLP2 may act as a neuroprotective agent.

## DISCUSSION

### Novel mitochondrial-derived peptides: communication molecules?

In addition to their well-characterized role as the “energy producers” of the cell, mitochondria are also critical for many aspects of cellular function, including apoptosis and regulation of oxidative stress. Mitochondria contain nearly 1,000 proteins, almost all of which are encoded by the nuclear genome. The mitochondrial chromosome has traditionally been thought to encode only 13 proteins that function primarily as components of the mitochondrial electron transport chain, along with specific rRNAs and 22 tRNAs. Here, we expand on a new paradigm in mitochondrial biology, previously proposed for the peptide HN, that was cloned in 2001 from mRNA transcribed from a region within mitochondrial 16S rRNA. In addition to the recently published MOTS-c peptide, the discovery of the SHLP peptides indicate that the mitochondrial genome may be full of yet-to-be-described sORFs coding for peptides that act as signaling molecules.

Our data unravel a novel function of the mitochondria: to produce specific MDPs that regulate cellular processes and human disease in an age-dependent manner. Specifically, age-dependent changes in the SHLP levels and other undiscovered MDPs might play a role in the development of age-related diseases. It is well recognized that multiple signals, mostly in the form of imported proteins, are delivered to the mitochondria to regulate its functions; however, the nature of mitochondrial retrograde signaling remains controversial. Although mitochondrial ROS or degraded protein products have been proposed to play such a role, especially in model organisms, they do not fully account for the complexity of tissue-specific mito-chondrial signaling in more complex organisms. We propose that mitochondria control the expression of MDPs that have specific activities in response to three major mitochondrial functions: apoptosis, metabolism, and oxidative stress. Our findings are consistent with recent reports [31, 32] that suggested that unknown factors termed ‘mitokines’ are produced from the mitochondria of *Caenorhabditis elegans* and secreted to communicate with other cells. Similar to HN, SHLPs may have both extracellular and intracellular effects, where the extracellular effects are mediated by a receptor that activates ERK and STAT-3 [47]. Further investigations into the extracellular and intracellular roles of SHLPs are required. Furthermore, although SHLPs 1–6 all originate from 16S rRNA, the various effects of individual SHLPs suggests that their expression is complex. We believe that each SHLP may activate its own unique receptor, leading to differential effects.

### Implications of SHLPs in cellular function in age-related diseases

SHLPs are derived from the mitochondrial genome within the 16S rRNA region, and we showed that they can regulate cell growth and function in a SHLP-specific manner (Fig. 2A–C). Like HN, SHLP2 and SHLP3 improved cell survival in response to toxic insults and prevented apoptosis, while SHLP6 had the opposite effect. Programmed cell death in response to extrinsic or intrinsic death signals is critical for tissue homeostasis. Age-related accumulations in cellular damage may lead to excessive cell death, limiting tissue function and life span. Increased apoptosis has been observed in neurodegenerative diseases such as AD, Parkinson's disease, and Huntington's disease [33]. Furthermore, SHLP2 and SHLP3 showed similar protective effects as HN, and significantly blocked cell death induced by STS (Fig. 3D). SHLP2 treatment alone also protected against Aβ_1–42_ induced cell death, which contributes to AD (Figs. 6B). Together this data suggests that SHLP2 and SHLP3 may participate in the pathogenesis of age-related neurodegenerative diseases.

### SHLP2 is a central and peripheral insulin sensitizer

HN is reportedly a central regulator of insulin action [10]. Continuous ICV infusion of HN significantly improved insulin sensitivity, and a single dose of the HN analogue HNGF6A significantly decreased blood glucose levels in Zucker diabetic rats. Similarly, in rats, continuous ICV infusion of SHLP2 significantly improved insulin sensitivity by increasing the GIR, suppressing HGP, and increasing peripheral glucose uptake in hyperinsulinemic-euglycemic clamp studies. This activity of SHLP2 is particularly significant because many centrally acting peptides that affect hepatic glucose metabolism (e.g., leptin, insulin, and IGF-I) have not been shown to increase peripheral glucose uptake. These results suggest that SHLP2 is an MDP that can communicate with the hypothalamus, under conditions that are yet to be determined, leading to a change in peripheral metabolism. In the future, SHLP2 may have a role in the treatment of diabetes because of its insulin-sensitizing effects.

### SHLP2 and SHLP3 regulate the expression of metabolic and inflammatory markers

Epidemiological studies have demonstrated that increased levels of mediators of inflammation and acute-phase reactants, such as fibrinogen, C-reactive protein (CRP), and IL-6, correlate with the incidence of type 2 diabetes mellitus (T2DM) [34–36]. In humans, anti-inflammatory drugs, such as aspirin and sodium salicylate, reduce fasting plasma glucose levels and ameliorate the symptoms of T2DM. In addition, anti-diabetic drugs, such as fibrates [37] and thiazolidine-diones [38], have been found to lower some markers of inflammation. SHLP2 increased the levels of leptin, which is known to improve insulin sensitivity, but had no effect on the levels of the pro-inflammatory cytokines IL-6 and MCP-1. SHLP3 significantly increased the leptin levels, but also elevated IL-6 and MCP-1 levels, which could explain the lack of an *in vivo* insulin-sensitizing effect of SHLP3. The mechanism by which SHLPs regulate the expression of metabolic and inflammatory markers remains unclear and needs to be further investigated. Furthermore, SHLPs have different effects on inflammatory marker expression, suggesting differential regulation and function of individual SHLPs.

### SHLP2 in aging

Mitochondria have been implicated in increased lifespan in several life-extending treatments [39, 40]; however, it is not known whether the relationship is correlative or causative [40]. Additionally, it is well known that hormone levels change with aging. For example, levels of aldosterone, calcitonin, growth hormone, and IGF-I decrease with age. Circulating HN levels decline with age in humans and rodents, specifically in the hypothalamus and skeletal muscle of older rats. These changes parallel increases in the incidence of age-associated diseases such as AD and T2DM. The decline in circulating SHLP2 levels with age (Fig. 6), the anti-oxidative stress function of SHLP2 (Fig. 3C), and its neuroprotective effect (Fig. 6B) indicate that SHLP2 has a role in the regulation of aging and age-related diseases.

### Conclusion

By analyzing the mitochondrial transcriptome, we found that sORFs from mitochondrial DNA encode functional peptides. We identified many mRNA transcripts within 13 protein-coding mitochondrial genes [1]. Such previously underappreciated sORFs have also been described in the nuclear genome [41]. The MDPs we describe here may represent retrograde communication signals from the mitochondria to the nucleus and may explain important aspects of mitochondrial biology that are implicated in health and longevity.

## METHODS

### Reagents

All peptides were synthesized by CPC Scientific (Sunnyvale, CA). The SHLP antibodies were custom synthesized by Harlan (Indianapolis, IN) and YenZym (San Francisco, CA); all other antibodies were purchased from Cell Signaling Technology (Danvers, MA). Western blotting reagents were purchased from Biorad (Hercules, CA). Cell culture reagents, primary cortical neurons, dihydroethidine (DHE), TRIzol^®^, Oil Red-O, and calcein-AM were purchased from Life Technologies (Grand Island, NY). Antibodies against phospho-STAT3 (Y705), total STAT3, phospho ERK-1/2 (T202/Y204), and total ERK-1/2 were purchased from Cell Signaling Technology. LINCOplex™ was purchased from Millipore (Billerica, MA). All primers and chemicals were purchased from Sigma (St Louis, MO). Luminescent ATP assay kits were purchased from Promega (Madison, WI).

### Cell culture

All cell lines (22RV1, NIT-1 murine β-cells, and 3T3-L1 murine pre-adipocytes) were purchased from ATCC (American tissue culture collection) (Manassas, VA). The human prostate carcinoma cell line 22RV1 was maintained in RPMI 1640 medium supplemented with 10% fetal bovine serum (FBS) and 1% penicillin/streptomycin. NIT-1 murine β-cells were cultured in F-12K medium supplemented with 10% FBS and 1% penicillin/streptomycin. 3T3-L1 murine pre-adipocytes were maintained in DMEM supplemented with 10% calf serum and 1% penicillin/streptomycin. For individual experiments, cells were seeded at a density of 1 × 10^5^ cells/cm^2^ in individual wells of 96-, 6-well plates, or 10-cm plates and grown to 80% confluence in a humidified atmosphere of 5% CO_2_ at 37°C before treatment. All treatments were carried out as indicated in serum-free media.

HeLa-ρ0 cells were derived from parental HeLa cells by culturing HeLa cells in the presence of 100 ng/mL ethidium bromide (EtBr) for more than 20 generations in DMEM [42].

### Cell death, proliferation, and viability assays

NIT-1 β-cells and 22Rv1 cells were cultured in complete media with control or SHLP peptides. To assess cell viability, cells growing in 96-well plates were analyzed using a CellTiter 96^®^ AQ_ueous_ One Solution MTS Assay kit (Promega) following the manufacturer's instructions. In 24-well plates, apoptosis was assessed by cell death detection using an ELISA (enzyme-linked immuno-sorbent assay) (Roche, Branchburg, NJ) according to the manufacturer's instructions. The cell proliferation rate was assessed by BrdU incorporation using ELISA (Roche) according to the manufacturer's instructions [43].

### Rabbit anti-SHLP1-6 antibody generation

Custom rabbit anti-SHLP 1, 3, and 5 were ordered from Yenzym and anti-SHLP 2, 4, and 6 were ordered from Harlan. High-titer polyclonal anti-sera against SHLPs 1–4 and 6 were obtained. Anti-sera against SHLP5 could not be generated due to technical issues.

### Mitochondrial extraction

PC3 cells were maintained in RMPI 1640 media with 10% FBS and subjected to RNA or mitochondrial isolation when the cell confluency reached 70–80%. The mitochondria were prepared from 4 × 10^8^ cells grown in 150-mm dishes. Cells were scraped in PBS (phosphate buffered saline), centrifuged (500 *g* for 5 min at 4°C), and then resuspended in 10 mL of ice-cold 1× MS (mannitol sucrose) homogenization buffer (210 mM mannitol, 70 mM sucrose, 5 mM Tris-HCl [pH 7.5], and 1 mM EDTA (ethylenediaminetetraacetic acid) [pH 7.5]). Cells were then homogenized 15 times using a 5-mL Teflon homogenizer, and the degree of homogenization was checked under a microscope. Homogenized cells were centrifuged (1,300 *g* for 5 min at 4°C) to sediment the nuclei. The centrifugation step was repeated to ensure that all nuclei were in the pellet fraction. Mitochondria were isolated from the supernatant by centrifugation (15,000 *g* for 15 min at 4°C), washed again by resuspending the pellet in 1× MS buffer, and centrifuged at 7,000–17,000 *g*.

### RNA isolation and Northern blotting

Total cell RNA and mitochondrial RNA were extracted using the TRIzol^®^ method followed by column purification (Direct-zol™ RNA MiniPrep; Zymo Research, Irvine, CA). mRNA was further purified by polyA selection using a Dynabeads^®^ mRNA Purification Kit (Thermo Scientific, Waltham, MA). The RNA concentration and purity were checked using a NanoDrop™ (Thermo Scientific) and agarose gel electrophoresis. Then, 130 ng of each RNA sample was separated on a 10% precast polyacrylamide tris-borate-EDTA(TBE)-urea gel (Bio-Rad, Hercules, CA) and transferred onto a positively charged nylon transfer membrane (Ambion^®^; Life Technologies). The blot was hybridized with 200 ng/mL digoxigenin (DIG)-labeled SHLP6 RNA probe in Easy Hyb™ hybridization buffer (Roche) for 16 h at 60°C. After two stringent washes in 2× SSC (saline sodium citrate buffer) (1× SSC contains 0.15 M NaCl and 0.015 M sodium citrate)-0.1% sodium dodecyl sulfate (SDS) for 5 min at room temperature and 0.2× SSC-0.1% SDS for 15 min at 60°C, the membrane was incubated for 30 min at room temperature with an anti-DIG antibody conjugated to alkaline phosphatase (Roche). Subsequent visualization was achieved using the BCIP-NBT substrate as instructed using a nucleic acid detection kit (Roche).

### Western blotting

Cell lysates were separated by SDS-PAGE (polyacrylamide gel electrophoresis) on tris-tricine or TGX^TM^ gels and transferred to polyvinyl-idenedifluoride membranes (Bio-Rad). Membranes were blocked in 0.2% I-Block (Applied Biosystems, Foster City, CA) in PBS containing 0.1% Tween 20 followed by incubation with the appropriate primary and secondary antibodies. Antibody-antigen complexes were visualized using a ChemiLucent ECL detection system (Millipore), followed by autoradiography.

### RT-PCR

Highly purified mRNA was isolated from 22Rv1 cells using TRIzol^®^ (Life Technologies), and PCR amplification was performed for SHLP 1–6 sORFs using unique primers (Supplemental Table S1) designed to amplify both mitochondrial and nuclear isoforms. The resulting PCR products were then analyzed by sequencing (Laragen, Venice, CA). Although these primers specifically targeted the appropriate strand of the mitochondrial transcript, due to possible self-priming and other technical reasons, the primers may have amplified mRNA from the opposite strand.

### Aβ_1–42_ induced cytotoxicity in neurons

The effect of SHLP2 and SHLP3 on the survival of mouse primary cortical neurons was assessed with LDH cytotoxicity assays (Thermo Scientific) following the manufacturer's instructions. Cells were treated with a control vehicle, 10 μM Aβ_1–42_ alone, or 10 μM Aβ_1–42_ in combination with 1 nM, 100 nM, or 10 μM SHLP2 or SHLP3. Supernatant (50 μL) was added to a 50-μL reaction mixture and incubated at room temperature for 30 min. The reaction was stopped with stop solution and the absorbance was measured at 490 nm.

### Adipocyte differentiation and Oil Red-O staining

3T3-L1 pre-adipocyte differentiation was induced by culturing cells for 2 days in growth media with 1 μM dexamethasone, 500 μM isobutylmethylxanthine, and 100 nM insulin, as previously described [44]. Cells were then cultured for 5 days in adipocyte differentiation media (growth media with 100 nM insulin). Throughout the 7 days of differentiation, cells were treated with 100 nM control peptide, SHLP2, or SHLP3, and the media was replaced every 2 days.

A 0.5% stock solution of Oil Red-O was prepared in 60% (v/v) isopropanol and filtered. For staining, cells were washed in PBS, fixed in 3.7% formaldehyde for 1 h, and stained with Oil Red-O for 1 h. Cells were photographed after washing twice with water.

### OCR and ATP production

The OCR was assessed using a Seahorse XF24 Extracellular Flux Analyzer as previously described [45]. ATP was measured in cells growing in 96-well plates using a CellTiter-Glo^®^ Luminescent Cell Viability Assay kit (Promega) following the manufacturer's instructions.

### ROS measurement

Intracellular ROS production was monitored through the conversion of the oxidant-sensitive dye DHE (Invitrogen) to fluorescent ethidium [46]. After peptides were treated in SF (serum free) media as indicated, NIT-1 or 22Rv1 cells were incubated with 10 μM DHE in Hanks' Balanced Salt Solution (HBSS) containing calcium, magnesium, and glucose for 30 min at 37°C. Fluorescence images were taken with an inverted fluorescence microscope (IX70; Olympus, Tokyo, Japan) at 15× magnification using the appropriate filters for ethidium fluorescence. Approximately 10 non-overlapping images from each culture dish were taken and cells were quantified using Photoshop (Adobe, San Jose, CA). Only cells whose fluorescence exceeded twice the basal level of fluorescence of non-treated cells were used for DHE fluorescence quantification.

### Systemic pancreatic insulin clamp and physiologic hyperinsulinemic-euglycemic clamp studies in rat

Control, SHLP2, or SHLP3 peptides were infused intracerebrally at a rate of 0.16 μg/kg/min into conscious SD rats. Glucose fluxes were studied under systemic pancreatic insulin and physiologic hyperinsulinemic-euglycemic clamp conditions. The GIR, hepatic glucose production, and peripheral glucose uptake were assessed as previously described [10]. The study protocol was reviewed and approved by the Institutional Animal Care and Use Committee of the Albert Einstein College of Medicine.

### Metabolic and inflammatory biomarkers

C57BL/6 mice (Jackson Laboratories, Bar Harbor, ME) were treated with 2 mg/kg/dose SHLP2 or SHLP3 BID for 5 days. Body weight, blood glucose, and food intake were measured daily. Mice were sacrificed and plasma was collected for analysis of the presence of metabolic (insulin and leptin) and pro-inflammatory (IL-6, MCP-1) biomarkers by LINCOplex™ (Millipore) following the manufacturer's instructions.

### SHLP2 levels in plasma

Plasma was collected from young (3 months old) and old (18 months old) male and female C57BL/6 mice (10 per group; National Institute of Aging, Bethesda, MD) in EDTA-containing Vacutainer^®^ tubes (BD, Franklin Lakes, NJ).

Endogenous plasma SHLP2 levels were measured using our developed SHLP2 ELISA using total immunoglobulin (Ig)G and ligand affinity purified antibodies with a detection limit of 50 pg/mL. The intra- and inter-assay coefficient variations (CV) of the SHLP2 ELISA were less than 10%.

Prior to the SHLP2 ELISA, from each sample, 100 μL of plasma was extracted using an acid solution (90% acetonitrile and 10% 1 N HCl). The supernatant was dried using a SpeedVac™(Thermo Fisher, Waltham, MA). The dried samples were reconstituted with assay buffer (50 mM PBS containing 0.5% Tween 20). Ninety-six-well microtiter plates were coated with SHLP2 capture antibody at a concentration of 0.5 μg/well in 200 μL of 50 mM sodium bicarbonate buffer (pH 9.5). The plates were incubated for 3–4 h at room temperature on a shaker, washed with wash buffer, and then washed twice with Superblock™ buffer (Pierce Chemicals, Rockford, IL). Standards, controls, or extracted samples were added to the appropriate wells with pre-titered detection antibody and incubated overnight. After washing, streptavidin-HRP (horse radish peroxidase) was added and further incubated for 30 min at room temperature. After four washes with wash buffer, 200 μL/well of OPD (o-phenylenediamine dihydro-chloride) substrate (1 mg/mL in hydrogen peroxide) was added and incubated for 10–20 minutes. Reactions were terminated with 50 μL/well 2 N H_2_SO_4_, and absorbance values were measured on a plate spectrophotometer (Molecular Designs, Sunnyvale, CA) at 490 nm.

### Statistical analysis

All values shown are presented as mean ± SEM (standard error of the mean). Independent two-tailed *t*-tests were used to compare the differences between two groups for analysis, such as cell viability, apoptosis, and proliferation. P < 0.05 was considered statistically significant.

## SUPPLEMENTAL DATA FIGURE AND TABLE


