# Supplementary material for: Naturally occurring mitochondrial-derived peptides are age-dependent regulators of apoptosis, insulin sensitivity, and inflammatory markers
Source: Aging (Albany NY). 2016 Apr 10;8(4):796–808. doi: 10.18632/aging.100943 (PMC4925829; doi:10.18632/aging.100943)
Supplement: Supplementary file 1 [file aging-08-796-s001.pdf]

SUPPLEMENTARY DATA

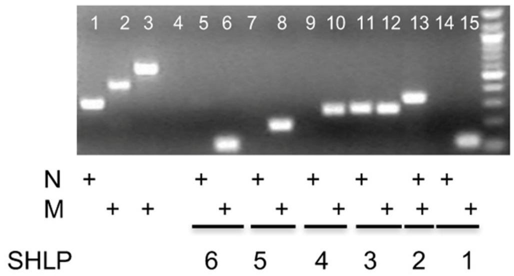

Figure S1. PCR amplification of SHLPs.

Table S1. Primer sequences for PCR of SHLP mRNA

Peptide	Primer Sequence
GAPDH	Forward: ACCACAGTCCATGCCATCAC<br>Reverse: TCCACCACCCTGTTGCTGTA
CO1	Forward: ATCCTACCAGGCTTCGGAAT<br>Reverse: CATCGGGGTAGTCCGAGTAA
16sRNA	Forward: CACTGTCAACCCAACACAGG<br>Reverse: GGCAGGTCAATTTCACTGGT
SHLP1	Mitochondria:<br>Forward: AAATCTTACCCCGCCTGTTT<br>Reverse: ACCTTTGCACGGTTAGGGTA<br>NUMT:<br>Forward: AAATCTTACCCCGCCTGTTT<br>Reverse: CGTATTCTCCACAGCCCCTA
SHLP2	Mitochondria and NUMT:<br>Forward: TGGTGATAGCTGGTTGTCCA<br>Reverse: AGGCTTATGCGGAGGAGAAT
SHLP3	Mitochondria:<br>Forward: ATTGAAACCTGGCGCAATAG<br>Reverse: TGGACAACCAGCTATCACCA<br>NUMT:<br>Forward: ATTGTAAACCGGCGCAATAG<br>Reverse: TGGACAACCAGCTATCACCA
SHLP4	Mitochondria:<br>Forward: GCATAAGCCTGCGTCAGATT<br>Reverse: CTACCTTTGCACGGTTAGGG<br>NUMT:<br>Forward: TAGACAAACCGGTGCTACCC<br>Reverse: CGTATTCTCCACAGCCCCTA
SHLP5	Mitochondria:<br>Forward: CCTAACAAACCCACAGGTCCT<br>Reverse: GGATTGCGCTGTTATCCCTA<br>NUMT:<br>Forward: CATTCAAGGGCATGTGGTTT<br>Reverse: GGGGACTTTAACACCCCACT
SHLP6	Mitochondria:<br>Forward: ACCTCGATGTTGGATCAGGA<br>Reverse: CCTGGATTACTCCGGTCTGA<br>NUMT:<br>Forward: CCAACCTCCGAACAACCTAA<br>Reverse: GGGCCCTATTTCTCTTGTCC
